# The lipid story in chronic kidney disease: a long story with a happy end?

**DOI:** 10.1007/s11255-012-0296-8

**Published:** 2012-10-02

**Authors:** Agata Kujawa-Szewieczek, Andrzej Więcek, Grzegorz Piecha

**Affiliations:** Department of Nephrology, Endocrinology and Metabolic Diseases, Medical University of Silesia, ul. Francuska 20-24, 40-027 Katowice, Poland

**Keywords:** Dyslipidemia, Chronic kidney disease, Cardiovascular disease, Statins

## Abstract

Cardiovascular (CV) morbidity and mortality increase with the severity of kidney disease, reaching 30 times higher mortality rates in dialysis patients compared with the general population. Although dyslipidemia is a well-established CV risk factor in the general population, the relationship between lipid disorders and CV risk in patients with chronic kidney disease (CKD) is less clear. Despite the clear evidence that statins reduce the risk of atherosclerotic events and death from cardiac causes in individuals without CKD, the use of statins in patients with kidney disease is significantly less frequent. For a long time, one of the explanations was the lack of a prospective, randomized, controlled study designed specifically to CKD patients. After recent publication of the data from Study of Heart and Renal Protection trial, given the safety and potential efficacy of statins, this lipid-lowering treatment should be administered more frequently to individuals with CKD stage 1–4, as well as those undergoing dialysis.

## Introduction

Cardiovascular disease (CVD) has been identified as the main cause of death among patients with end-stage renal disease (ESRD). It has been reported that the cardiovascular (CV) mortality is up to 30 times higher in dialysis patients than in the general population [1]. Moreover, there is evidence that cardiovascular events occur more frequently already at early stages of chronic kidney disease (CKD) and the cardiovascular mortality increases with the severity of kidney disease [2]. Altogether, patients with CKD stage 3 and 4 are more likely to die of cardiovascular causes than to reach ESRD requiring renal replacement therapy [3].

Although dyslipidemia is a well-established cardiovascular risk factor in the general population, the relationship between lipid disorders and CV risk in patients with chronic kidney disease is less clear. The causes of lipid metabolism disorders in patients suffering from CKD are complex and depend on the degree of renal failure. In a cross-sectional study of over 16,000 patients from NHANES III population, lower GFR was associated with decreased apolipoprotein A-I (ApoA-I) and increased apolipoprotein B (ApoB) serum concentrations [4].

The role of HMG-Co-A reductase inhibitors (statins) in lowering the cardiovascular risk in the general population is well established. In a meta-analysis of randomized trials, statin therapy was calculated to reduce the risk of major coronary events, coronary revascularization, and stroke by approximately 20 % for each 1 mmol/L reduction in LDL cholesterol [5]. The efficacy and safety of statins at early stages of CKD have been shown in large prospective and retrospective studies [6–19].

According to the European Best Practice Guidelines (EBPG) [20] and American Kidney Disease Outcomes Quality Initiative (K/DOQI) guidelines [21], the presence of kidney disease should be considered as a major risk factor for atherosclerosis and CVD progression. Therefore, patients with CKD are potential candidates for treatment with lifestyle changes and lipid-lowering drugs, mainly statins.

## Lipid abnormalities at different stages of CKD

### Non-dialysis patients with CKD

#### Non-nephrotic CKD patients

The atherogenic lipid profile in patients with CKD is characterized by lower levels of high-density lipoproteins (HDL), higher triglyceride (TG), ApoB, lipoprotein(a) [Lp(a)], remnant intermediate (IDL), and very low-density lipoproteins (VLDL) as well as a greater proportion of oxidized low-density lipoproteins (oxLDL). The degree of disturbances in lipoprotein metabolism is associated with the rate of declining of glomerular filtration rate (GFR).

The main dyslipidemic disturbance observed in CKD patients is the elevated concentration of triglycerides and TG-rich VLDL and IDL remnants [22, 23]. Decreased activity of peripheral lipoprotein lipase (LPL) and hepatic lipase (HL) seems to be the main cause of these lipids abnormalities. One potential mechanism responsible for increased TG levels in CKD appears to be the elevated apolipoprotein C-III (ApoC-III) concentration, a direct inhibitor of LPL [24]. Moreover, several lines of evidence suggest a role for the hyperparathyroidism in the delayed catabolism of TG [25, 26]. Besides the impaired lipolysis, a downregulation of lipoprotein receptors may be involved in the development of dyslipoproteinemia in patients with chronic kidney disease. A linkage between decreased clearance of TG-rich lipoproteins and reduced expression of hepatic LDL receptor-related protein (LRP) and VLDL receptor has been found in experimental models of CKD [27, 28].

In patients with CKD, total and serum LDL cholesterol concentrations are usually within the target range or even lower. Additionally, the plasma concentrations of lipid subfractions may not fully reflect the CV risk. Patients with chronic kidney disease may have a greater proportion of oxidized LDL particles, which are recognized by scavenger receptors and induce the formation of foam cells in atherosclerotic plaques [29, 30]. Furthermore, LDL particles in CKD patients tend to be smaller and denser, therefore more atherogenic [31, 32].

Low serum HDL cholesterol concentration is observed in the majority of patients with kidney disease. It is most probably caused by a decreased activity of lecithin cholesterol acetyltransferase (LCAT), an enzyme involved in esterification of cholesterol and maturation of HDL from pre-β-HDL to HDL3 and HDL2 [33, 34]. Increased activities of cholesterol ester transfer protein (CETP) and acyl CoA: cholesterol acyltransferase (ACAT) are also related to the lower HDL concentration in CKD [35]. Moreover, patients with kidney disease have been shown to have lower plasma concentration of ApoA-I and ApoA-II due to reduced expression of these proteins in the liver [36]. The change in serum HDL concentration may also be affected by the presence of inflammation and decreased albumin concentration in patients with renal failure [22].

#### Nephrotic syndrome patients

Elevated serum concentrations of total and LDL cholesterol as well as increased serum triglycerides concentrations are typical lipid abnormalities in patients with nephrotic syndrome [37]. Both proteinuria and hypoalbuminemia stimulate the activity of 3-hydroxy-3-methylglutaryl CoA (HMG-CoA) reductase and ACAT [38], as well as may decrease the expression of the LDL receptor in the liver [39]. Impaired clearance of TG-rich lipoproteins [40] and elevated hepatic synthesis of VLDL [41] seem to be the main causes of hypertriglyceridemia in patients with massive proteinuria.

The results of HDL measurements in patients with nephrotic syndrome are inconsistent. Some authors have reported higher [42] levels of HDL cholesterol, while others have found lower [43] or normal [44] concentration of this lipoprotein in the presence of proteinuria.

### Dialysis-dependent CKD patients

Dyslipidemia in hemodialysis patients is characterized by hypertriglyceridemia, low serum HDL concentration, and usually normal levels of total and LDL cholesterol [45–47] similar to the non-dialysis population with CKD. It has been observed, however, that additional factors like repeated use of heparin [48] or type of membranes used [49] may affect lipid metabolism in these patients.

In the majority of published studies, peritoneal dialysis patients had more atherogenic lipid profile (higher total and LDL cholesterol, triglycerides and Lp(a) concentration, greater proportion of oxidized and small, dense LDL particles) than those on hemodialysis [31, 32, 50–52]. Two important factors may explain this phenomenon. First, glucose absorption from the dialysate solution and coexisting insulin resistance may promote TG synthesis through elevated availability of free fatty acids [53, 54]. Secondly, the loss of protein, including apolipoproteins, across the peritoneal membrane may lead to lipid abnormalities similar to those in the nephrotic syndrome [55].

## Lipid abnormalities in patients with CKD and cardiovascular disease

### Non-dialysis patients with CKD

#### Non-nephrotic CKD patients

Patients with chronic kidney disease have higher prevalence of both traditional and non-traditional CV risk factors than those with normal kidney function [56–58]. In a prospective cohort study of 17,898 participants, including 807 patients with CKD (estimated GFR between 15 and 59 mL/min/1.73 m^2^) followed for 10.5 years, Muntner et al. [4] evaluated the relationship between total cholesterol and TG levels and major coronary events. They found that participants with increased total cholesterol or triglyceride serum concentration suffered more coronary events irrespective of baseline eGFR. Moreover, lipoprotein(a) concentration and Lp(a) phenotype, especially small Lp(a), were described as important cardiovascular risk factors in non-nephrotic CKD patients [59, 60].

#### Nephrotic syndrome patients

So far, the association between the CV risk and nephrotic syndrome has been investigated only in small cohorts of patients, generating inconsistent results [61, 62]. Ordonez et al. [63] have found that the risk of myocardial infarction and death from coronary heart disease was, respectively, 5.5 (95 % CI 1.6–18.3) and 2.8 (95 % CI 0.7–11.3) times higher in patients with nephrotic syndrome (*n* = 142) than in the control group (*n* = 142). Contrary, other authors failed to confirm the association between the presence of nephrotic syndrome (steroid-responsive/dependent nephrotic syndrome during childhood) and an increased risk of CVD [64].

### Dialysis-dependent CKD patients

#### Hemodialysis patients

The CV mortality in hemodialysis patients is significantly higher than in the general population [56]. Although a positive association between total serum cholesterol level and the risk of CVD in the general population is well documented, studies in dialysis patients led to conflicting results [60, 65–69]. Moreover, some authors have observed an increased mortality among patients with lower cholesterol levels [70, 71]. This phenomenon, referred to as “reverse epidemiology” or “reverse causality,” may be explained as a result of confounding by other factors, specifically protein energy wasting and/or inflammation. Iseki et al. [68] followed 1,167 hemodialysis patients for 10 years and found that low cholesterol concentration was associated with higher mortality as well as with higher C-reactive protein (CRP) and lower serum albumin concentrations. However, in a subgroup of patients with serum albumin values ≥4.5 g/dL (*n* = 128), the adjusted hazard ratio was 1.37 (CI 95 % 1.10–1.69, *p* = 0.0034), suggesting that there is a positive relationship between the risk of death and the level of serum cholesterol in well-nourished patients. Other authors have supported this hypothesis, founding that hypercholesterolemia is a strong risk factor for CV mortality only in the absence of inflammation (CRP < 10 mg/L, IL–6 < 3.09 pg/mL) or malnutrition (albumin > 36 g/L) [72].

Kilpatrick et al. [70] followed a cohort of 15,859 maintenance hemodialysis patients for 3 years and noted that not only total hypercholesterolemia but also LDL hypercholesterolemia and hypertriglyceridemia tend to show a reverse association with survival.

The role of Lp(a), as an independent risk factor for CV mortality, is well documented [66, 73]. In a study of 390 patients on HD, the concentration of Lp(a) was two times higher in dialysis group than in healthy controls and was significantly associated with CV mortality [73].

#### Peritoneal dialysis patients

Peritoneal dialysis patients have a more atherogenic lipid profile than patients on HD. Therefore, it seems credible that the CV risk related to dyslipidemia may be greater in this group of patients. So far, the association between CVD and dyslipidemia in PD patients has been investigated in small cohorts, in a few retrospective studies [74–76]. Nevertheless, some authors have noted a similar correlation between a low cholesterol level and malnutrition [77, 78], as well as the elevated Lp(a) [79] and a higher mortality rate in this population.

## Lipid abnormalities and progression of CKD

In 1982, Moorhead et al. [80] hypothesized that progression of chronic kidney disease may be mediated by abnormalities in lipid metabolism. It has been suggested that hyperlipidemia as well as oxidized Lp(a) and LDL particles may play an important role in kidney injury [81, 82]. Although the potential mechanisms for these abnormalities are not completely elucidated, it seems credible that circulating LDL, by binding to the glycosaminoglycans in the glomerular basement membrane, may lead to an increased permeability of the membrane. Filtered lipoproteins may stimulate proliferation of mesangial cells and contribute to glomerular damage. Even though some of the filtered lipoproteins are reabsorbed in proximal tubules, the rest will be altered passing down the nephron and, if intraluminal pH is close to the isoelectric point of the apolipoprotein, it will precipitate causing tubulointerstitial damage [80, 83].

Attman et al. [84] observed an association between accumulation of triglyceride-rich apoB-containing lipoproteins and a more rapid loss of renal function in a group of patients with non-diabetic kidney disease. Other authors have noted that oxidized Lp(a) and LDL particles cause apoptotic cell death in the vascular wall and in the glomerulus through the stimulation of the O_2_ formation [81]. Moreover, it has been reported that oxidized LDL, but not native LDL, may induce the loss of nephrin (a principal component of the slit diaphragm) and apoptosis in human cultured podocytes, which may lead to proteinuria [82].

## Lipid-lowering therapy in pre-dialysis CKD patients

### Cardiovascular outcomes

The evidence that patients at early stages of CKD benefit from lipid-lowering therapy comes from meta-analyses and post hoc analyses of large cardiovascular statin trails in the general population [6–15, 87]. Tonelli et al. [6] analyzed the data from the Pravastatin Pooling Project (PPP) and showed that moderate CKD, defined by eGFR of 30–59.9 mL/min/1.73 m^2^, was independently associated with increased CV risk. Pravastatin significantly reduced the incidence of the primary outcomes (time to myocardial infarction, coronary death, or coronary revascularization) similarly in the subjects with normal kidney function (HR 0.78, 95 % CI 0.65–0.94) and in the subgroup of 4,491 patients with moderate CKD (HR 0.77, 95 % CI 0.68–0.86). Moreover, in patients with moderate CKD and high CV risk, pravastatin reduced all-cause mortality (HR 0.86, 95 % CI 0.74–1.00, *p* = 0.045). Therefore, the authors suggested that patients with impaired kidney function would benefit from statin therapy more than those with normal renal function [6]. In another post hoc analysis of data from a randomized trial of pravastatin 40 mg daily versus placebo, greater absolute risk reduction in the primary outcome (time to myocardial infarction, coronary death, or percutaneous/surgical coronary revascularization) was observed in patients with CKD (stages 2 and 3) or diabetes (6.4 %) than in individuals without any of these diseases (3.5 %) [7].

Sever et al. [8] in the Anglo-Scandinavian Cardiac Outcomes Trial (ASCOT) assessed benefits of statin therapy in primary prevention of coronary heart disease in patients with arterial hypertension and non-fasting total serum cholesterol concentrations ≤6.5 mmol/L. In a subgroup analysis of 6,517 subjects with kidney dysfunction, defined as serum creatinine concentration 130–200 μmol/L in males and 110–200 μmol/L in females, atorvastatin (10 mg/day) reduced the risk of non-fatal myocardial infarction and cardiovascular death (HR 0.61, 95 % CI 0.44–0.84). In 1,329 patients with impaired renal function (serum creatinine concentration 130–200 μmol/L in males and 110–200 μmol/L in females) from the 20,536 adults, who were enrolled into the Heart Protection Study (HPS), simvastatin use (40 mg/day) was associated with a significant reduction in major CV events [9].

Szummer et al. [10] analyzed data from the nationwide SWEDEHEART registry of over 42,000 patients following myocardial infarction and showed that statins use was associated with significant reduction in mortality at 1 year in patients with mild to severe chronic kidney disease, although the prescription of statins at discharge was much less frequent in patients with severe CKD (29 %) than in those with normal renal function (81 %).

A post hoc analysis of the Air Force/Texas Coronary Atherosclerosis Prevention Study assessed the efficiency of lovastatin in the prevention of first major acute CV event in participants with mild CKD, defined by eGFR < 60 mL/min/1.73 m^2^. A significant decrease in CV events such as fatal and non-fatal coronary events (RR 0.35, 95 % CI 0.13–0.93; *p* = 0.03), fatal and non-fatal CV events (RR 0.39, 95 % CI 0.16–0.93; *p* = 0.03), and coronary revascularization procedures (RR 0.23, 95 % CI 0.07–0.77; *p* = 0.01), was found in patients with renal dysfunction, even after adjustment for potential confounders [11].

Colhoun et al. [12], reporting data from the randomized placebo-controlled trial-Collaborative Atorvastatin Diabetes Study (CARDS), found that the risk of major CVD events and stroke decreased by 42 and 61 %, respectively, in the subgroup of 970 patients with CKD stage 3 (eGFR 30–60 mL/min/1.73 m^2^) treated with atorvastatin.

In a recently performed secondary analysis of the JUPITER (Justification for the Use of Statins in Prevention-an Intervention Trial Evaluating Rosuvastatin) trial, treatment with rosuvastatin (10 mg daily) was associated with a 45 % reduction in risk of myocardial infarction, stroke, or cardiovascular death (HR 0.55, 95 % CI 0.38–0.82; *p* = 0.002) and 44 % reduction in all-cause mortality (HR 0.56, 95 % CI 0.37–0.85; *p* = 0.005) in participants with moderate CKD [13].

Holdaas et al. [14] performed a pooled analysis of 30 double-blind, randomized trials to compare the efficacy of fluvastatin in persons with moderate to severe CKD (creatinine clearance < 50 mL/min) and patients with normal renal function or mild kidney dysfunction (creatinine clearance ≥ 50 mL/min). Fluvastatin compared to placebo reduced combined cardiac death and nonfatal myocardial infarction in both groups, in patients with moderate to severe renal insufficiency and in patients with normal renal function or mild renal insufficiency, by 41 and 30 %, respectively.

The PREVEND IT (Prevention of Renal and Vascular End Stage Disease Intervention Trial) is the only published randomized, double-blind, placebo-controlled trial performed to asses the role of statins in patients with microalbuminuria and mild CKD (eGFR <60 mL/min/1.73 m^2^). Contrary to previous findings, subjects treated with pravastatin had no significant risk reduction in the primary endpoints (cardiovascular mortality and hospitalization for cardiovascular morbidity) after 4 years (*p* = 0.64) [85], as well as in extended 10-year follow-up (*p* = 0.99) [86].

The Cholesterol Treatment Trialists’ (CTT) Collaboration [15] reported a meta-analysis of data from randomized trials involving at least 1,000 participants and at least 2-year treatment duration of LDL-cholesterol-lowering therapy versus control. There was no evidence for reduction in major CV events (major coronary events, stroke, or coronary revascularization) in patients with eGFR <30 mL/min/1.73 m^2^, although such a reduction was observed in patients with CKD stages 2 and 3.

A Cochrane Collaboration summarized 26 studies (25,017 participants) to evaluate the effects of statins in pre-dialysis CKD. Statins significantly decreased total and LDL cholesterol, as well as the risk of all-cause (RR 0.81, 95 % CI 0.74–0.89) and cardiovascular mortality (RR 0.80, 95 % CI 0.70–0.90) in this group of patients [87].

The Assessment of LEscol in Renal Transplantation (ALERT) trial [88] compared fluvastatin (*n* = 1,050) with placebo (*n* = 1,052) in patients after renal transplantation. After a mean follow-up of 5 years, fluvastatin failed to impact the composite primary endpoint, defined as cardiac death, non-fatal myocardial infarction, or coronary intervention procedure (*p* = 0.139). However, there was a slight reduction in cardiac death and non-fatal myocardial infarction in the group treated with fluvastatin (RR 0.65, 95 % CI 0.48–0.88; *p* = 0.005). During a 2-year extension of the ALERT trial, 1,652 renal transplant recipients who received fluvastatin demonstrated a lower risk of major cardiac events, cardiac death, or non-fatal myocardial infarction [16]. Moreover, a post hoc analysis has suggested a beneficial effect of early introduction of lipid-lowering therapy in patients after kidney transplantation: When fluvastatin treatment was initiated at 0–2 versus >6 years after transplantation, the observed frequency of cardiac death and non-fatal MI was 3.2 versus 8.2 %, respectively [17]. A possible impact of statins on patient and graft survival was evaluated among 2,041 first-time renal allograft recipients also by Wiesbauer et al. [18]. The authors observed that statin use was associated with reduced all-cause mortality (adjusted HR 0.64, 95 % CI 0.48–0.86; *p* = 0.003) and prolonged patient survival, while no effect on graft survival was noted.

Only a few lines of evidence suggest a role for other lipid-lowering agents in the prevention of cardiovascular disease in the CKD population. A post hoc subgroup analysis of the randomized double-blind, placebo-controlled Veterans’ Affairs High-Density Lipoprotein Intervention Trial (VA-HIT) was performed to evaluate the cardiovascular benefits of gemfibrozil in patients with creatinine clearance ≤75 mL/min/1.73 m^2^ [89]. Treatment with gemfibrozil was associated with a lower rate of cumulative incidence of major cardiovascular events (nonfatal myocardial infarction, fatal coronary disease, or stroke) in this subgroup (adjusted HR 0.75, 95 % CI 0.59–0.96). There was, however, higher rate of non-coronary death and increase in the serum creatinine concentration in gemfibrozil-treated participants.

Finally, the SHARP (Study of Heart and Renal Protection) trial [19] was created to assess the beneficial effect of the combination of simvastatin plus ezetimibe in patients with CKD and no history of myocardial infarction or coronary revascularization (Table 1). Of the total 9,270 participants, 6,247 were not on dialysis at the beginning of the study. They were initially randomized to receive simvastatin (20 mg) plus ezetimibe (10 mg), simvastatin (20 mg), or placebo. There was no difference in the incidence of adverse events between simvastatin alone and simvastatin plus ezetimibe group, and after the first year of observation, patients who initially received simvastatin were re-randomized to simvastatin plus ezetimibe and placebo groups. The median follow-up was 4.9 years, and the main outcome was the first major atherosclerotic event, defined as non-fatal myocardial infarction, coronary death, non-hemorrhagic stroke, or arterial revascularization procedure (excluded non-CHD cardiac death and hemorrhagic stroke). On average, there was a difference in LDL cholesterol of 0.85 mmol/L and a significant 17 % reduction in major atherosclerotic events (RR 0.83, 95 % CI 0.74–0.94; *p* = 0.0021) as well as in major vascular events (RR 0.85, 95 % CI 0.77–0.94; *p* = 0.0012) in patients treated with simvastatin plus ezetimibe. The use of simvastatin plus ezetimibe had no effect on vascular mortality, but the coronary heart disease was a cause of death only in 181 patients in this study. During the follow-up, there was no evidence that this combination therapy increased the risk of cancer, myopathy, or hepatitis in CKD patients.

**Table 1:** The effects of lipid-lowering therapy on cardiovascular outcomes in CKD patients

Study	Population	Duration (years)	Causes of death	Treatment	LDL cholesterol reduction	Primary endpoint	Secondary endpoint
4D [101]	1,255 HD patients with DM type 2	4	CHD 9 % Stroke 6 % Sudden death 26 % Heart insufficiency 6 % Other CV 3 % Non-CV 6 %	Atorvastatin 20 mg/day	42 %^a^	Composite of non-fatal MI or cardiac death (fatal MI, sudden death, death due to congestive heart failure, death after interventions to treat CHD, other death due to CHD) and non-fatal or fatal stroke HR 0.92, 95 % CI 0.77–1.11	Death from all causes RR 0.93, 95 % CI 0.79–1.08 All cardiac events combined (death from cardiac causes, non-fatal MI, CABG, PTCA) RR 0.82, 95 % CI 0.68–0.99 All cerebrovascular events combined (ischemic, hemorrhagic or other stroke, TIA, PRIND) RR 1.12, 95 % CI 0.81–1.55
AURORA [102]	2,776 HD patients	3.8	CHD 10.8 % Stroke 2.7 % Other cardiac 2.4 % Other vascular 3.3 % Other CV 0.04 % Non-CV 18.6 %	Rosuvastatin 10 mg/day	43 %^b^	Composite of non-fatal MI or cardiac death, non-fatal or fatal stroke and other vascular death HR 0.96, 95 % CI 0.84–1.11	Death from all causes HR 0.96, 95 % CI 0.86–1.07 Death from non-cardiovascular causes HR 0.92, 95 % CI 0.77–1.09 Major cardiovascular event or cause-specific death HR 0.94, 95 % CI 0.84–1.05 Atherosclerotic cardiac event (CHD and nonfatal MI) HR 0.96, 95 % CI 0.81–1.14 Vascular access procedure HR 1.10, 95 % CI 0.95–1.27 Revascularization HR 0.98, 95 % CI 0.78–1.23
SHARP [19]	9,270 CKD patients: 6,247 not on dialysis 2,527 on HD 496 on PD	4.9	CHD 1.95 % Stroke 1.6 % Other cardiac 3.7 % Other vascular 0.8 % Sudden death 1.1 % Nonvascular 13.8 %	Simvastatin 20 mg/day plus ezetimibe 10 mg/day	77 %^c^	Composite of MAEs: RR 0.83, 95 % CI 0.74–0.94 Non-fatal MI RR 0.84, 95 % CI 0.66–1.05) Coronary death RR 1.01, 95 % CI 0.75–1.35 Non-hemorrhagic stroke RR 0.75, 95 % CI 0.60–0.94 Arterial revascularization RR 0.79, 95 % CI 0.68–0.93	Composite of MVEs: RR 0.85, 95 % CI 0.77–0.94 MAEs RR 0.83, 95 % CI 0.74–0.94 Other MVEs (non-coronary cardiac deaths and hemorrhagic strokes) RR 0.94, 95 % CI 0.78–1.14

*HD* hemodialysis, *PD* peritoneal dialysis, *DM* diabetes mellitus, *LDL* low-density lipoprotein, *CKD* chronic kidney disease, *CHD* coronary heart disease, *CV* cardiovascular, *MI* myocardial infarction, *MAE* major atherosclerotic event, *MVE* major vascular event, *CABG* coronary artery bypass graft, *PTCA* percutaneous transluminal coronary angioplasty, *TIA* transient ischemic attack, *PRIND* prolonged reversible ischemic neurologic deficit

^a^After 4 weeks of treatment

^b^After 3 months of treatment

^c^After 8–13 months of treatment

In conclusion, there is evidence suggesting that patients with CKD stages 2 and 3 may benefit similarly or even more from statin therapy than those with normal renal function. Most of this evidence, however, comes from meta-analyses and post hoc analyses of trails in the general population and not from prospective, randomized, controlled study designed specifically for CKD population. After publication of the SHARP trial results, the US Food and Drug Administration (FDA) approved the combination of simvastatin plus ezetimibe as lipid-lowering treatment routinely used in kidney disease patients. The K/DOQI guidelines suggest that patients with pre-dialysis CKD should be treated according to the NCEP/ATP III guidelines recommended for the general population [21].

### Renal outcomes

In the last two decades, the nephroprotective effects of statins have been evaluated in numerous experimental models as well as in retrospective and prospective studies (Table 2).

**Table 2:** The effects of lipid-lowering therapy on renal outcomes in CKD patients

Study	Population	Duration	Treatment	Renal outcomes
Abe et al. [96]	91 patients with CKD stages 1–3	24 weeks	Rosuvastatin	Parameters at baseline and after 24 weeks of treatment: Serum creatinine (mg/dL) 1.07 ± 0.04 (at baseline) and 1.07 ± 0.05 (after 24 weeks of treatment); *p* = NS eGFR (ml/min/1.73 m^2^) 55.1 ± 2.1 (at baseline) and versus 55.1 ± 2.1 (after 24 weeks of treatment); *p* = NS Cystatin C (mg/L) 1.08 ± 0.04 (at baseline) and versus 1.03 ± 0.04 (after 24 weeks of treatment); *p* < 0.0001 Urinary albumin/creatinine ratio (mg/g Cr) 308 ± 38 (at baseline) versus 195 ± 25 (after 24 weeks of treatment); *p* < 0.0001
Bianchi et al. [97]	56 patients with CKD	1 year	Atorvastatin	Parameters at baseline and after 1 year of treatment: Urine protein excretion (g/day) 2.2 ± 0.1 (at baseline) versus 1.2 ± 1.0 (after 1 year of treatment); *p* < 0.01 Creatinine clearance (ml/min) 51 ± 1.8 (at baseline) versus 49.8 ± 1.7 (after 1 year of treatment); *p* = NS
Baigent et al. [19]	6,247 patients with CKD not on dialysis at randomization	4.9 years	Simvastatin plus ezetimibe	Main renal outcome: ESRD (the need of dialysis or transplantation) RR 0.97, 95 % CI 0.89–1.05; *p* = NS Tertiary renal outcomes: ESRD or death RR 0.97, 0.90–1.04; *p* = NS ESRD or doubling of baseline creatinine RR 0.93, 0.86–1.01; *p* = 0.09

*CKD* chronic kidney disease, *eGFR* estimated glomerular filtration rate, *ESRD* end-stage renal disease

A protective role of rosuvastatin against puromycin and adriamycin-induced p21-dependent apoptosis in mouse podocytes has been reported by Cormack-Aboud et al. [90], suggesting that statins may decrease proteinuria and delay the progression of CKD. Moreover, statins may exert anti-proteinuric effects through the stimulation of Akt activity and inhibition of the oxLDL-induced apoptosis and loss of nephrin [82]. Fluvastatin significantly decreased urinary albumin excretion and glomerular sclerosis, as well as increased nephrin expression in podocytes in a murine model of HIV-associated nephropathy [91].

In the post hoc analysis of the Greek Atorvastatin and Coronary Heart Disease Evaluation (GRACE) study, patients with coronary heart disease and dyslipidemia treated with atorvastatin (*n* = 783) had a 12 % increase in creatinine clearance [92]. This effect appeared to be dose-related and was more pronounced among participants with early stages of CKD. This observation has been confirmed in the subanalysis of the Treating to New Targets (TNT) study, in which a significant difference in mean change from baseline eGFR at the end of the follow-up was noted between patients with coronary heart disease treated with atorvastatin in dose 10 mg (*n* = 4,829; increase of 3.5 ± 0.14 mL/min/1.73 m^2^) and 80 mg daily (*n* = 4,827; increase of 5.2 ± 0.14 mL/min/1.73 m^2^) [93]. Tonelli et al. [94] have reported that compared to placebo, pravastatin reduced the rate of renal function decline in 1,702 patients with moderate CKD but did not reduce the risk of a ≥25 % decline of eGFR. In a subsequent meta-analysis of 27 randomized, controlled trials, statin therapy was associated with reduced rate of decline in eGFR (1.22 mL/min/year slower) compared to placebo [95]. However, this effect was significant only in the CVD subpopulation but not in participants with glomerulonephritis, diabetes mellitus or hypertension. Navaneethan et al. [87] in a Cochrane database review did not confirm the beneficial effects of statins on eGFR but found statins reduced urinary protein excretion in six studies. Similarly, in two small, prospective, controlled open-label studies rosuvastatin [96] and atorvastatin [97] decreased proteinuria in patients with CKD. Finally, the SHARP trial showed no protection from reaching ESRD in 6,247 participants not on dialysis at the beginning of the study [19] (Table 2).

## Lipid-lowering therapy in dialysis-dependent CKD patients

Studies in patients with end-stage renal disease demonstrated the effectiveness of statins in lowering LDL cholesterol and similar incidence of adverse events as in patients without ESRD. The use of statins had been associated with reduced total and cardiovascular mortality in observational studies. Large randomized controlled trials, however, surprisingly failed to confirm the benefits in dialyzed patients (Table 1).

Seliger et al. [98] performed a secondary analysis of the data from the United States Renal Data System Dialysis Morbidity and Mortality Study Wave 2 (USRDS DMMS-2) finding that treatment with a statin was associated with lower risk of total (adjusted RR 0.68, 95 % CI 0.53–0.86; *p* = 0.002) and cardiovascular mortality (adjusted RR 0.63, 95 % CI 0.44–0.91; *p* = 0.014) both in patients on hemodialysis and peritoneal dialysis. This observation was confirmed by Mason et al. [99], who analyzed data from the Dialysis Outcomes and Practice Patterns Study (DOPPS) and showed a 23 % reduction in the risk of cardiovascular deaths and a 44 % reduction in the non-cardiac mortality among HD patients treated with statins. In a subsequent observational study, the prescription of statins was associated with a 41 % lower risk of all-cause mortality in peritoneal dialysis patients [100].

The 4D Study (Deutsche Diabetes Dialyse Studie) was a randomized, double-blind, placebo-controlled study in 1,255 patients with type 2 diabetes mellitus and dialysis-dependent CKD who were randomized to receive 20 mg atorvastatin (*n* = 619) or placebo (*n* = 636) (Table 1). A 42 % reduction in the median LDL cholesterol concentration was observed, to approximately 70 mg/dL after 4 weeks of lipid-lowering therapy. Although the risk of all cardiac events combined (death from cardiac causes, nonfatal myocardial infarction, coronary artery bypass graft, percutaneous transluminal coronary angioplasty) was reduced by 18 % in the atorvastatin group (RR 0.82, 95 % CI 0.68–0.99; *p* = 0.03), the cumulative incidence of the primary endpoint (CV death, nonfatal MI or stroke) did not differ significantly between atorvastatin and placebo groups (HR 0.92, 95 % CI 0.77–1.11; *p* = 0.37). It is of note that coronary heart disease was a cause of death only in 9 %, while sudden death, heart failure, and other cardiovascular disease accounted for 35 % deaths. The incidence of fatal stroke was unexpectedly higher in the atorvastatin group compared with placebo (27 vs. 13); however, the prevalence of combined cerebrovascular events was similar between both groups [101].

Similar results were found in the following randomized, double-blind, placebo-controlled study—AURORA (A Study to Evaluate the Use of Rosuvastatin in Subjects on Regular Hemodialysis: An Assessment of Survival and Cardiovascular Events) (Table 1) [102]. 2,776 patients treated with regular hemodialysis or hemofiltration for ≥3 months were randomized to receive rosuvastatin 10 mg daily (*n* = 1,391) or placebo (*n* = 1,385). The mean follow-up was 3.2 years, and the primary endpoint was defined as the time to a major cardiovascular event (nonfatal myocardial infarction, nonfatal stroke, or death from cardiovascular causes). There was a reduction in the LDL, total cholesterol, and triglycerides concentrations, by 42.9, 26.6, and 16.2 % respectively, in the rosuvastatin group. No significant differences in the primary cardiovascular endpoint were observed between the rosuvastatin and placebo groups (HR 0.96, 95 % CI 0.84–1.11; *p* = 0.59). Moreover, there was no association between the primary endpoint and the LDL cholesterol level initially and after treatment. Nevertheless, a post hoc analysis of the diabetic participants of the AURORA trial have shown that treatment with rosuvastatin reduced their atherosclerotic coronary events by 32 % (HR 0.68, 95 % CI 0.51–0.90; *p* = 0.008) [103].

In 9,270 participants of the SHARP trial, 33 % were dialysis-dependent at randomization (27 % on hemodialysis and 5 % on peritoneal dialysis) (Table 1). The reduction in LDL cholesterol level in dialysis patients (0.6 mmol/L) was smaller compared to pre-dialysis ones (0.96 mmol/L). Moreover, in this subgroup, the baseline LDL concentration (2.6 vs. 2.9 mmol/L) and the use of lipid-lowering therapy (54 vs. 65 %) were lower than in non-dialysis-dependent CKD patients. However, as the authors pointed out, the SHARP study had no sufficient statistical power to compare dialysis and pre-dialysis subjects. Given the overall beneficial effect of simvastatin plus ezetimibe on the risk of major atherosclerotic events in the studied population, it may be concluded that these results are relevant to all patients with CKD [19]. Further studies are needed to investigate the association between the use of statins and cardiovascular mortality in dialysis patients in particular.

One of the possible explanations for the lack of beneficial cardiovascular effects of statins in dialysis patients may be a different pathology of arterial wall in this group. Non-traditional risk factors (uraemia-specific), such as hyperhomocysteinemia, chronic fluid volume overload, anemia, increased calcification and microinflammatory state, might be responsible for higher incidence of CVD among ESRD subjects [104, 105]. Vascular calcification is found as arterial intima calcification in atherosclerotic plaques (related to classical CV risk factors) or arterial media calcification (related to calcium/phosphorus disorders and the duration of HD). It has been documented that this non-occlusive arterial media calcification is one of the main, independent predictors of all-cause and CV mortality in dialysis patients [106]. Moreover, in the ESRD population, sudden death is more frequent than atherosclerotic cardiac events and the mechanism of sudden death is only partially dependent on the atherosclerotic coronary artery disease. Left ventricular hypertrophy and remodeling comprising fibrosis and capillary rarefaction, heart failure due to fluid volume overload, and electrolyte abnormalities causing arrhythmias may be the main causes of sudden death in this group of patients [107]. Therefore, according to Holdaas et al. [103], the selected primary endpoints in outcome studies should share a common pathophysiology potentially modifiable by the evaluated treatment.

In summary, having in mind the safety of statins presented in 4D and AURORA studies and after recent publication of the data from the SHARP trial, this lipid-lowering treatment should be administered more frequently to individuals with CKD stages 1–4, as well as to those undergoing dialysis. Although atherosclerosis may not be the most frequent cause of death in patients with impaired kidney function, statins still reduce the risk of atherosclerotic events in this population contributing to better survival.
